# A call for equity: Investing in substance use research for Brazil’s LGBTQ+ population

**DOI:** 10.1016/j.clinsp.2025.100790

**Published:** 2025-09-29

**Authors:** Clarice Sandi Madruga, Katia Isicawa de Sousa Barreto, Danilo Seabra, Claudio Jerônimo da Silva, Guilherme Sabino de Godoy Filho, Carmen Silvia Molleis Galego Miziara, Martha Canfield, Quirino Cordeiro, Ronaldo Ramos Laranjeira

**Affiliations:** a Department of Psychiatry, Universidade Federal de São Paulo (UNIFESP), São Paulo, SP, Brazil; b Faculdade Paulista de Ciências da Saúde, Medicine Development São Paulo’s Association (SPDM), São Paulo, SP, Brazil; c Faculdade de Medicina da Universidade de São Paulo, São Paulo, SP, Brazil; d Department of Psychology, Glasgow Caledonian University, Glasgow, United Kingdom; e Faculty of Medical Sciences of Santa Casa de São Paulo, São Paulo, Brazil

Dear Editor,

We write to urge the readership of the Clinicis to support and advocate for increased investment in research on substance use within the LGBTQ+ community in Brazil. This diverse population includes individuals who identify as Lesbian, Gay, Bisexual, Transgender, Genderqueer (LGBTQ+) as well as non-binary, agender, asexual, or another sexual or gender identity that is either or both non-heterosexual or noncisgender (defined as someone whose gender aligns with traditional norms related to the sex they were assigned at birth). In line with the 2024 Revised Declaration of Helsinki^1^ which emphasises the inclusion of marginalised populations, including LGBTQ+ people in health research, there is a pressing need for inclusive and equitable research environments. Advancing this agenda is especially critical in low- and middle-income countries such as Brazil, where public policies and cultural attitudes toward LGBTQ+ people are varied and often shaped by inequality, social exclusion, and discrimination^2^

Despite recent efforts, epidemiological research on substance use prevalence, its consequences, and associated mental health comorbidities among LGBTQ+ populations in Brazil remains in its infancy. A national household survey of 85,859 adults found significantly higher rates of hazardous alcohol use, daily tobacco consumption, and polysubstance use among LGBTQ+ individuals compared to their heterosexual peers^3^ Gay men were nearly twice as likely as heterosexual men to engage in polysubstance use, while lesbian women were almost three times as likely to report heavy drinking compared to heterosexual women. A cross-sectional study in Fortaleza with 254 LGBTQ+ adults reported substance use rates of 91.5 % for alcohol, 67.4 % for tobacco, and 66.9 % for cannabis, with 9.8 % assessed as high risk for dependence^4^ Findings from the 2012 Brazilian National Alcohol and Drugs Survey also revealed that individuals identifying as sexual minorities were more likely to experience alcohol-related problems (i.e., alcohol dependence and binge drinking) as well as to use crack cocaine and hallucinogens, compared to heterosexual individuals^5^ However, critical research gaps remain. These include a lack of longitudinal data examining the links between substance use and mental health outcomes, particularly concerning depression and suicide, which are disproportionately prevalent among LGBTQ+ populations globally^6^ Transgender and non-binary individuals remain underrepresented in both clinical and epidemiological studies, which often prioritise HIV-related risks while neglecting broader substance use contexts. Additionally, practices like chemsex (use of substances to facilitate/enhance sexual activity) remain poorly understood in Brazil despite being commonly reported in cross-sectional surveys among the LGBTQ+ population, with prevalence rates ranging from 29.0 % to 36.6 %^7^^,^^8^ The social and structural factors that drive engagement in chemsex remain largely underexplored, limiting effective responses in both research and policy^7^

Another urgently needed yet underexplored area of research is on the LGBTQ+ people who frequent Open Drug Scenes (ODS). These scenes are commonly defined as urban areas with high concentrations of drug use and trafficking. ODS are widespread across major cities in Brazil, and they are closely linked to severe social vulnerability, embedded poverty, and limited access to public health services. Unlike many other countries where opioid use predominates in such contexts, smoked crack cocaine is the primary substance consumed in Brazil’s ODS. Since 2016, the Survey of Drug Use Scenes in Capitals (Levantamento de Cenas de Uso em Capitais – LECUCA), systematically monitored population size, demographics, health status, and substance use patterns in São Paulo’s ODS, with Brasilia and Fortaleza joining the initiative in 2022^9^ According to LECUCA, LGBTQ+ people accounted for 7.5 % of participants in 2019 (18/240), 3.17 % in 2022 (18/568), and 5.67 % in 2024 (8/141). A particular concerning trend observed by the last three survey waves is the escalating rate of physical violence experienced by LGBTQ+ individuals within ODS: from 44.44 % in 2019 to 55.55 % in 2022, and a striking 87.50 % in 2024. In addition, reports of early-life adverse experiences among LGBTQ+ participants have also increased. In 2022, 44.44 % of LGBTQ+ participants reported having experienced abuse during childhood, up from 27.78 % in 2019. This pattern of violence exposure is not limited to ODS. A recent analysis of data from the Ministry of Health’s Notifiable Diseases Information System (SINAN), carried out by the São Paulo Association for the Development of Medicine (SPDM) in partnership with the São Paulo Public Prosecutor’s Office (MPSP), revealed a 1200 % increase in the number of LGBTQ+ individuals receiving care through Brazil’s Unified Health System (SUS) in the state of Sao Paulo following experiences of violence (rising from 1063 cases in 2014 to 13,804 in 2023)^10^ For cases where the perpetrator was reported, approximately 8 % involved the victim’s parents^10^ While it is unclear whether this increase reflects improved reporting or a rise in actual incidents, SINAN and LECUCA data underscore the persistently high rates of violence experienced by the LGBTQ+ population in Brazil.

Exposure to violence, particularly in childhood, is a well-documented long-term driver of homelessness among LGBTQ+ people^11^ Once unhoused, these people face heightened vulnerability to further violence and problematic drug use^11^ In Brazil, the majority of people who use drugs in ODS are homeless/street homeless[9] and represent some of the most marginalised and socially excluded populations in the country. They are subject to overlapping forms of stigma, poverty, and institutional neglect, which severely limit their access to support services^12^ This persistent cycle of marginalisation is further intensified by state-led crackdowns (government-initiated police and municipal actions that frequently involve violence, forced removal, and mass arrests to dismantle drug use hotspots, control public space, and assert state authority over ODS populations). While many high-income countries have seen gradual reductions in homophobia and anti-LBGTQ+ violence^13^ Brazil continues to grapple with deeply rooted hostility toward LGBTQ+ communities. Legally, the country offers one of the most comprehensive frameworks in LGBTQ+ rights in Latin America, including marriage equality, legal gender recognition, and criminalisation of homophobia and transphobia^14^ However, these legal advancements have not been matched by broader social acceptance. Structural discrimination and widespread social prejudice perpetuate a climate of violence, social exclusion, and systemic inequality^2^ In this context, LGBTQ+ individuals in ODS face compounded risks to their physical and mental health, safety, and survival.

These intersecting challenges demand not only urgent and inclusive public health interventions but also a robust research agenda to guide them. There is a pressing need for empirical studies that examine the unique experiences of LGBTQ+ people with substance use problems, including those in ODS, with particular attention to the impacts of violence, trauma, homelessness, and drug use across the life course. This includes longitudinal research, qualitative studies centring lived experiences, evaluations of different types of treatment and support models tailored to LGBTQ+ needs. Investment in such research is essential to inform policies and programmes that are not only inclusive in name but effective in practice, grounded in evidence, and responsive to the realities of those most at risk.

## Declaration of competing interest

The authors declare no conflicts of interest.
